# Metastasis to the Male Breast from Squamous Cell Lung Carcinoma

**DOI:** 10.1155/2013/593970

**Published:** 2013-08-13

**Authors:** Berhan Genç, Aynur Solak, Neslin Şahin, Aşkın Gülşen

**Affiliations:** ^1^Department of Radiology, Şifa University School of Medicine, Izmir, Turkey; ^2^Department of Chest Diseases, Şifa University School of Medicine, Izmir, Turkey

## Abstract

Metastasis to breast from extra mammarian organs is quite rare with an incidence of 0.5–3%. Malignancies that most commonly metastasize to breast are lymphomas, leukemias, and malignant melanoma. Metastasis of lung cancer to breast is a very rare condition. We present here a case with squamous cell lung cancer that metastasized to breast. A 65-year-old man presented with cough in addition to a mass in the left breast, which had been noted 3 weeks ago and grown gradually since then. A histopathological diagnosis of metastasis of squamous lung cancer was made for the mass in the left breast. PET/CT scan showed no distant metastasis. Chemoradiation therapy was applied for lung cancer. As the prognosis of such patients is extremely poor, it is of a great importance to distinguish a primary breast cancer from a metastatic breast lesion in order to determine the appropriate treatment modality.

## 1. Introduction

Although breast cancer is prevalent among adult females, metastasis to breast tissue from an extra mammarian malignancy is quite rare. The incidence has been reported to be 0.5–3% [1, 2]. Despite its rare occurrence, breast metastasis is treated way differently than primary breast cancer. Although malignancies metastasizing to breast vary considerably, diseases that most commonly metastasize to breast are melanoma, leukemia, lymphoma, and renal adenocarcinoma [1, 3]. Patients are usually females. Malignancies metastasizing to breast from extra mammarian organs are extremely uncommon in men. PET/CT is highly useful for detection of metastatic lesion. Definite diagnosis is made by histopathological examination. In this case report, a primary lung cancer that metastasized to the breast was presented with imaging findings. To our knowledge, this case is the first male patient diagnosed with simultaneous squamous cell lung cancer and breast metastasis presented by PET/CT.

## 2. Case Presentation

**Figure 1:**
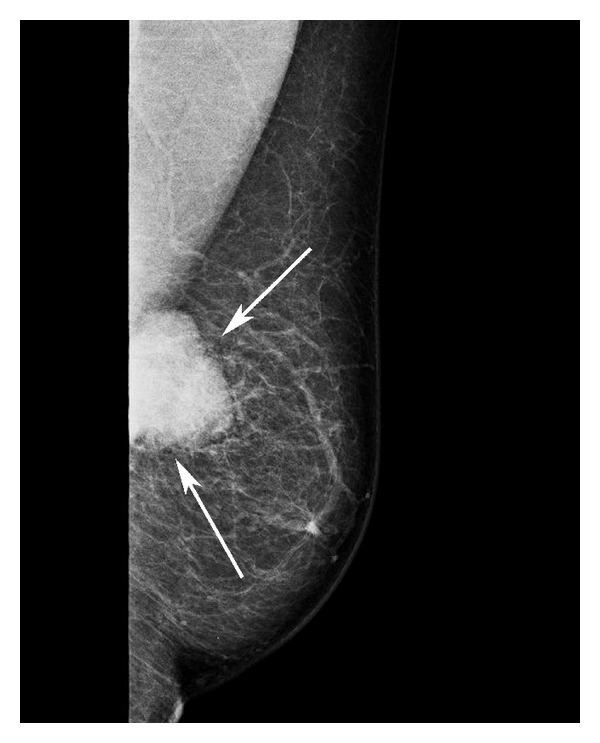
Lateral oblique mammography of the left breast shows smooth-bordered, big soft tissue lesion (arrows) at the upper middle quadrant.

**Figure 2:**
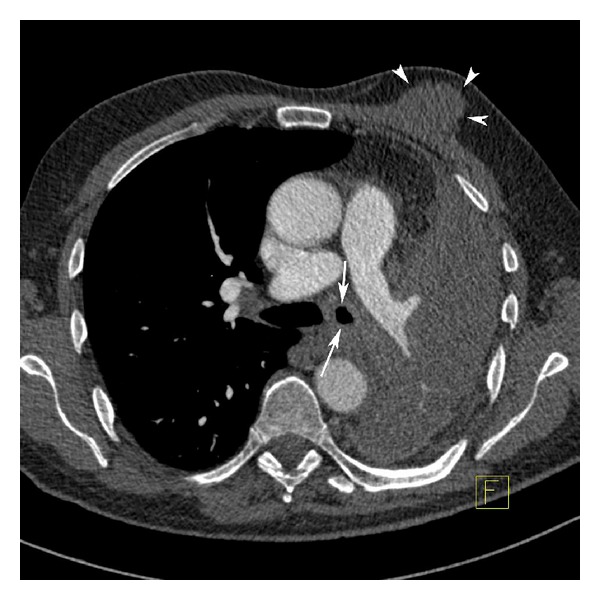
Thoracic CT shows massive pleural effusion and the left main bronchial obliteration (arrows) in the left lung. CT also shows the solid mass (arrowheads) in the left breast.

**Figure 3:**
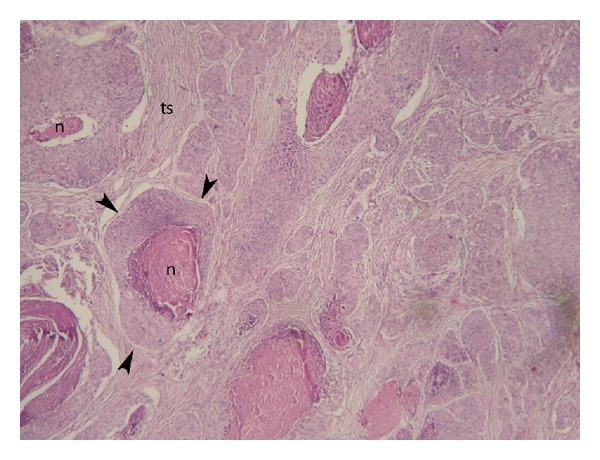
Microphotograph shows tumor islets (arrowheads) containing a central area of necrosis formed by atypical squamous cells as well as keratin globules H&E ×40. n: necrosis, ts: tumor stroma.

A 65-year-old male patient presented with firm nodule in the left breast and cough for 3 weeks. On chest examination, respiratory sounds diminished in the left hemithorax. Physical examination revealed a painless, well-bordered, and firm mass lesion at the upper middle quadrant of the left breast. No palpable lymph nodes were present in the left axilla. Breast ultrasonography revealed a well-bordered, solid mass lesion. Color Doppler interrogation showed a high-resistance arterial vascular flow inside the mass. Mammography revealed a 5 × 4 cm mass lesion at the upper middle quadrant of the left breast (Figure 1). Chest X-ray was notable for pleural effusion involving the entire left hemithorax. Chest computed tomography demonstrated massive pleural effusion compressing the entire left lung. Prevascular area contained multiple lymphadenopathies of which the biggest one had a diameter of 2 cm. A mass with hazy borders that invaded left main bronchus was detected at the left infrahilar region. A solid mass with a smooth contour was also found in the left breast tissue (Figure 2). A Tru-cut breast biopsy was performed to differentiate a primary breast lesion from a metastatic one. The breast biopsy was consistent with metastasis of squamous cell carcinoma. Microscopically, tumor islets formed by atypical squamous cells as well as keratin globules were noted inside a fibrous stroma with no ductus formation (Figure 3). Immunohistochemical studies revealed p63(+) tumor cells. Estrogen receptors (ER) were progesterone receptors (PR), and thyroid transcription factor-1 (TTF-1) were all negative.

**Figure 4:**
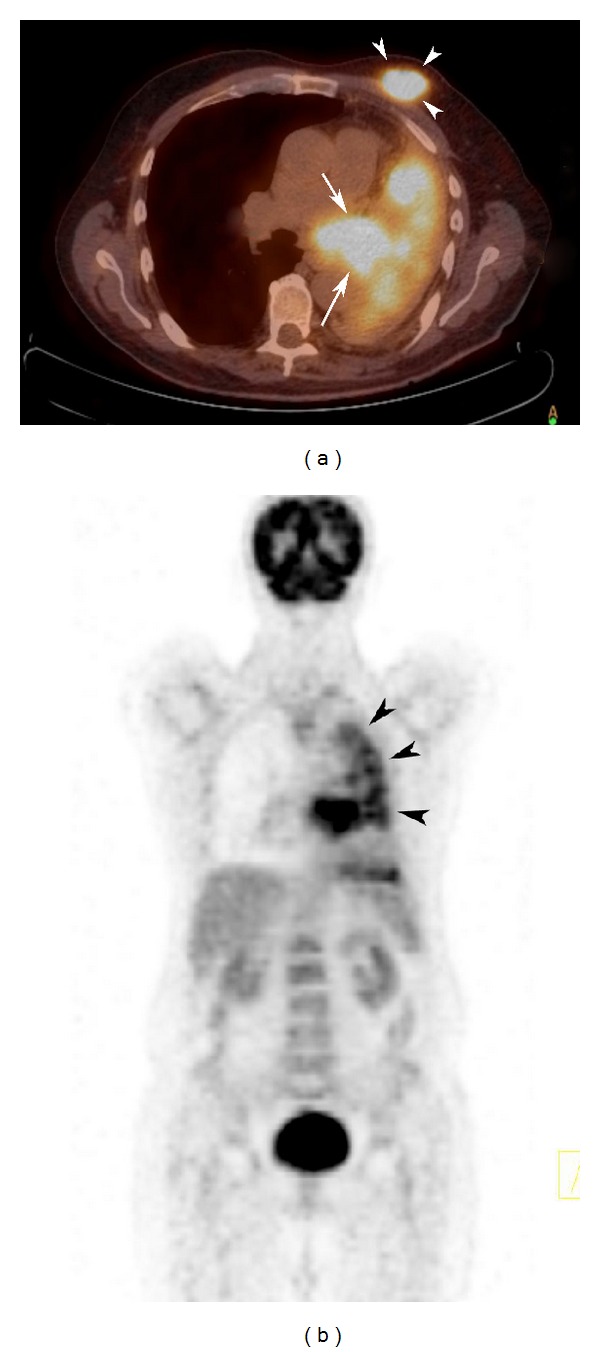
PET/CT demonstrates a primary lung cancer (arrows) at left hilar region and hypermetabolic masses with a high FDG uptake in left breast (arrow heads), consistent with metastasis (a). Whole body PET/CT was not positive for an FDG uptake suggesting metastasis in an organ other than the left breast. Nodular multiple high FDG uptake at the lateral part of the left lung was considered a false positive pleural uptake (arrow heads) (b).

 Bronchoscopy showed a mass lesion 1.5 cm distal to the left main bronchus, which completely invaded and obstructed the left main bronchus. Histopathological examination of the bronchoscopic material revealed a primary squamous cell carcinoma. PET/CT for staging of the primary malignancy demonstrated hypermetabolic masses with high FDG uptake in the left hilar region and in the left breast that were considered to represent a primary lung cancer and a breast metastasis, respectively (Figure 4). Also, multiple nodular areas of pleural origin with a high FDG uptake were detected at the lateral part of the left lung. Pleural biopsy was consistent with pleuritis. Cytologic examination of the pleural fluid was consistent with empyema. Multiple pleural FDG uptake was considered a false finding.

The stage of the disease was T3 N2 M1, stage IV. Concurrent chemoradiation therapy 4 courses of etoposide with cisplatin and external irradiation to the mediastinum and tumor site were given. Unfortunately, the patient died 6 months after the diagnosis. 

## 3. Discussion

Male breast cancer constitutes only 1% of all breast cancers and 1% of all male malignancies [4, 5]. Breast metastases from extra mammarian organs are responsible for 0.5–3% of all breast malignancies. Breast metastasis of lung cancer is very rare. Lung cancer most commonly metastasizes to brain, bone, liver, and surrenal glands. Other less common sites of metastasis are stomach, pancreas, small intestine, choroid plexus, muscles, umbilicus, and penis. Breasts are metastasis site of hematological malignancies, melanoma, renal cell carcinoma, ovarian cancer, thyroid cancer, and carcinoid tumors of small intestine [1, 3, 6–8]. To our knowledge, breast metastasis of squamous lung cancer is very rare. The majority of patients are females while male breast metastases are extremely rare. Metastatic masses of the breast may progress rapidly to reach a considerable size, as in our patient [9]. 

Mammography may be useful in differentiating primary and metastatic breast malignancies. As in our case, the typical mammographic presentation of metastatic malignancies are round, dense masses [10]. Microcalcification and spiculation in mass contour are quite rare with an exception for metastases of ovarian cancer. In addition, no skin thickening or distortion is found, because metastatic breast mass causes minimal proliferation of the fibrous tissue surrounding the lesion, and the mass is of the same size in mammography and palpation [11–13]. Primary breast cancer, on the other hand, is bigger than its mammographic size [14]. In ultrasonography, in contrast to a primary breast cancer, the metastatic mass appears as a hypoechoic, well-bordered mass lesion that does not lead to distortion in adjacent structures [15].

PET/CT shows abnormal metabolic activity in a tissue and provides quantitative and qualitative metabolic information about the tumor. This modality is used for staging of primary breast cancer; however, it is not recommended for diagnosis of primary breast cancer [16]. In our patient, PET/CT revealed only the left breast metastasis of the primary lung cancer. Primary and metastatic cancers of the breast show high FDG uptake on PET/CT. However, a false FDG uptake may also be observed in case of postoperative changes, acute and chronic inflammations of breast, lactation, fat necrosis, and benign lesions of breast such as fibroadenoma [17].

Although primary and metastatic breast cancers have basic differences in imaging findings, they appear surprisingly similar. Tru-cut biopsy is quite efficient in demonstration of primary and metastatic diseases in breast tissue [18]. As in our case, it may be needed for confirmation of diagnosis with immunohistochemical studies. 

As a conclusion, in males, secondary metastatic breast cancer is extremely uncommon. From the viewpoint of the treatment approaches, metastatic breast cancer should not be confused with primary breast cancer. Even when a male presents with a mass in breast, the possibility of metastasis should be remembered to provide early diagnosis and optimal therapy.
